# Looking for a Novel Vegan Protein Supplement from Faba Bean, Lupine, and Soybean: a Dietary and Industrial Standpoint

**DOI:** 10.1007/s11130-023-01125-y

**Published:** 2023-12-07

**Authors:** Hend A. Hamed, Walaa Kobacy, Elsayed A. Mahmoud, Mennatallah M. A. El-Geddawy

**Affiliations:** 1 Faculty of Agriculture, Horticulture Department, Sohag University, Sohag, 82524 Egypt; 2 Faculty of Agriculture, Food Science & Nutrition Department, Sohag University, Sohag, 82524 Egypt; 3 Faculty of Agriculture, Food Science & Technology, Department, Assuit University, Assuit, 71526 Egypt

**Keywords:** Complementary nutrition, Dietary supplement, Plant-based protein, Plant macromolecules

## Abstract

Global population growth poses a threat to sustainable development. Meanwhile, the use of plant proteins as healthy and sustainable alternatives to animal proteins needs further research. Therefore, this investigation was designed to study the nutritive, structural, and thermal properties of isolated protein fractions from different legumes, *i.e*., faba bean (FPI), soybean (SPI), and lupine (LPI). As a prospective plant-based protein powder, an equal mixture (MPI) of the three prior legume samples was formulated to study its properties compared to each sole sample. The alkaline extraction and isoelectric precipitation (AE-IP) technique was used for protein isolation. Results showed that all protein isolates had reasonable levels of protein with maximum protein content in SPI (96.15%). The MPI sample, however, came out on top in terms of amino acid profile followed by FBI. Compared to SPI and LPI, it had the highest isoleucine content and higher methionine, valine, leucine, phenylalanine, and lysine. Moreover, MPI showed a median particle charge (−37.1 mV) compared to FPI, SPI, and LPI samples. MPI sample peak showed resistance to heat denaturation at a temperature greater than 200 °C when the DSC test was conducted. With respect to its rheological characteristics, it outperformed the other three protein isolates and exhibited the highest values of storage modulus G’ and loss modulus G”. Consequently, our study suggests that pulse-derived protein isolate mixture can be used as a unique type of nutritious dietary protein supplement. It could be a good nutritional alternative to proteins derived from animals.

**Supplementary Information:**

The online version contains supplementary material available at 10.1007/s11130-023-01125-y.

## Introduction

There is a high prevalence of protein malnutrition in developing nations, and by 2050, there may be a demand for up to 50% more protein-rich foods [1]. To date, animal-based protein is still regarded as a reliable source of protein because it provides a significant amount of essential amino acids, is easier to digest, and has higher functional properties than other protein sources. However, plant-based proteins are now the way to improve human health and lower the environmental footprint. Legumes are a perfect source of proteins and have a balanced nutritional profile. The potential to use numerous legume types in different forms to create novel foods is a growing trend today that meets consumer demand [2, 3]. Due to their high level of adaptability to various climatic and environmental conditions as well as the fact that they support ecosystem services through biological nitrogen fixation and crop diversity, legumes are recognized as sustainable crops. One of the earliest legume crops grown worldwide is faba bean (*Vicia faba* L.) [4], which is a rich source of minerals, vitamins, carbohydrates, lysine, and several bioactive substances. Lupine (*Lupinus termis*) and soybean (*Glycine max*) seeds are two legume crops that are low in carbohydrates, high in protein, and contain oil [5, 6]. In a thorough investigation of customers who had nutritional deficiencies or would like to get their full food ration, many visited pharmacies to obtain nutritional supplements. Doctors also prescribe these supplements for athletes, patients, or malnourished individuals to meet their daily requirements, maintain better health, or even grow muscles. It is becoming increasingly prevalent to deliver protein in various products, such as protein shakes, bars, and powders. Protein powders are simple to incorporate into a range of foods and beverages such as yogurt, juice, or tea. Commercial protein powders are found in the form of protein concentrates, which have more carbs and fat than protein isolates [7]. They are mostly dependent on dairy products such as whey protein, and some people experience lactose intolerance symptoms when consuming them. Additionally, soybeans are widely available as a vegan choice and an inexpensive source of protein powders [8, 9]. A new generation of products based on legumes such as peas or faba beans started to get a decent turnout. Some people, however, fear that these types lack certain nutrients. According to Boukid & Castellari [10], the application of faba bean proteins is still limited due to their low functional properties. Meanwhile, Gorissen [11] suggested that combining several plant-based protein sources may result in proteins with properties that closely resemble those of animal proteins. In addition to the balanced amino acid profile consumers seek to obtain from their protein products, food companies have long prioritized certain criteria, *i.e*., particle size, rheological behavior, viscosity, as well as stable structures across a wide temperature range for market expansion. Consequently, there is a strong need to highlight all these factors. Therefore, our objective was to produce a nutritional and functional plant-based protein supplement from a mixture of three legume protein isolates; faba beans, lupines, and soybeans compared to a single plant protein and provide the same targeted properties as animal proteins. This formula might work well as a substitute for animal-based protein.

## Materials and Methods

The Materials and Methods section is presented as supplementary material.

## Results and Discussion

### Total Protein Content

**Fig. 1:**
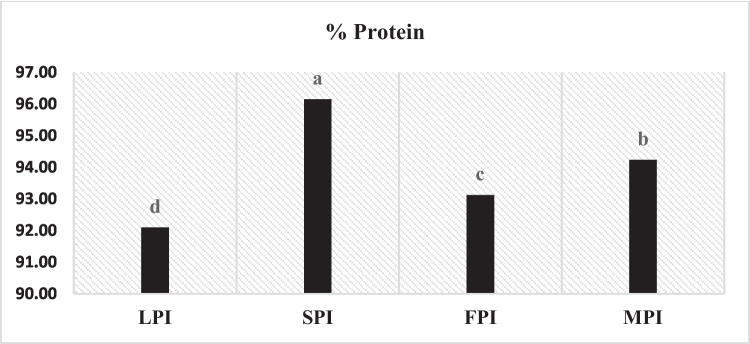
Total protein content of protein isolates derived from lupine (LPI), soybeans (SPI), faba bean (FPI), and a mixture of the three isolates (MPI)

Figure 1. depicts that all examined protein isolates from lupine, faba, and soybeans, as well as the combination of the three isolates, had a decent amount of protein ranging between 92.10- 96.15, with a significant difference *p* < 0.05 among them. The findings were consistent with Eckert et al. [12] who found that the protein content of faba bean protein isolates ranges between 88 and 94%. According to research by Felix et al. [13], it was found that utilizing the isoelectric precipitation method to extract proteins has significant benefits over other approaches such as acid extraction or dry fractionation since it yields a larger amount of protein (60%) with a better extraction efficiency.

### Amino Acid Profile

Any substitute protein must provide the same balanced amino acids as animal proteins. Legumes are used to improve the protein content in a variety of food items due to their high amino acid levels. In this framework, a thorough investigation of the amino acid profile was performed in our research work, in which 17 amino acids of FPI, SPI, LPI, and MPI were identified (Table 1). These include essential and nonessential amino acids. The FPI, SPI, LPI, and MPI amino acid profiles showed significant variations.

**Table 1:** Amino acid composition of protein isolates derived from faba bean, soybeans, lupine, and a mixture of the three isolates (mg g^−1^ protein)

Amino acids	FPI	SPI	LPI	MPI
Met+Cys	11.4	3.46	2.03	8.42
Val	89.3	19.6	8.30	50.9
Ileu	85.9	24.3	9.60	87.2
Leu	42.1	34.0	18.4	35.2
Phe	84.5	30.7	18.6	47.1
Lys	41.8	25.4	11.8	35.9
His	60.7	21.6	9.10	62.6
Thr	33.6	21.6	13.4	45.0
Asp	73.1	99.1	61.7	114
Glu	53.2	125	57.2	90.3
Ser	37.9	35.9	19.2	52.4
Gly	19.3	20.0	10.5	27.8
Arg	100.6	45.4	25.0	84.9
Ala	25.5	16.1	12.0	37.1
Pro	24.8	51.2	10.5	42.4
Tyr	105.1	27.3	10.1	71.6

*FPI* Faba bean protein isolate, *SPI* Soybean protein isolate, *LPI* Lupine protein isolate, *MPI* Mixture protein isolate, *Met* methionine, *Cys* cyctein, *Val* valine, *Ileu* isoleucine, *Leu* leucine, *Phe* phenylalanine, *Lys* lysine, *His* histidine, *Thr* threonine, *Asp* aspartic acid, *Glu* glutamic acids, *Ser* serine, *Gly* glycine, *Arg* arginine, *Ala* alanine, *Pro* prolamine, *Tyr* tyrosine

From Table 1, LPI contained adequate levels of methionine+cyctein (Met+Cys), serine (Ser), aspartic (Asp), glutamic acids (Glu), and arginine (Arg). Our findings concurred with Klupšaitė & Juodeikienė [14]. Compared to LPI, the SPI sample had more amino acids but fewer than FPI and MPI. The profile we found in our research is comparable to what Kudeka and colleagues [15] found for soy protein in their investigation. Meanwhile, the SPI’s amino acids levels discovered by Mohsen et al. [16] were greater than ours. The soil type, planting date, and fertilizer treatment may be partially responsible for the discrepancy between our data and their findings. Furthermore, according to several scholars including Nosworthy et al. [17], the amounts of amino acids differ significantly between varieties. Interestingly, the most crucial amino acids, valine (Val), methionine (Met), phenylalanine (Phe), histidine (His), lysine (Lys), leucine (Leu), isoleucine (ILeu), and threonine (Thr) were present in significant concentrations in the FPI sample. While Ayala-Rodríguez [18] and other researchers found that Glutamic, Histidine and threonine were the highest amino acids content when compared with the amino acid composition of faba bean protein flour. Methionine and cysteine (Met+Cys), two necessary sulfur-containing amino acids, are frequently limiting factors in protein quality. Rushovich and Weil [19] found that sulfur fertilization enhanced Met +Cys production in soybeans and therefore protein quality. The increases in Met+Cys in the protein were 71 and 79%, respectively. The most noteworthy observation was that after using an equal amount of LPI, SPI, and FBI to make the MPI sample, it surpassed the two sole protein isolates (LPI and SPI) in all amino acids except proline and glutamic acid which were higher in the soybean protein isolate. The amino acid content of MPI was comparable to that of the FPI isolate in the essential amino acids; the sulfur containing amino acids; (Met+Cysteine), and the essential and non-essential amino acids; Val, Phe, Lys, leu, Ileu, His, Thr, Ser, Glu, Asp, Proline (Pro), and Alanine (Ala). We could infer that mixing different sources of legume protein isolate samples was in favor of MPI’s amino acids content.

### Zeta Potential (ζ)

**Fig. 2:**
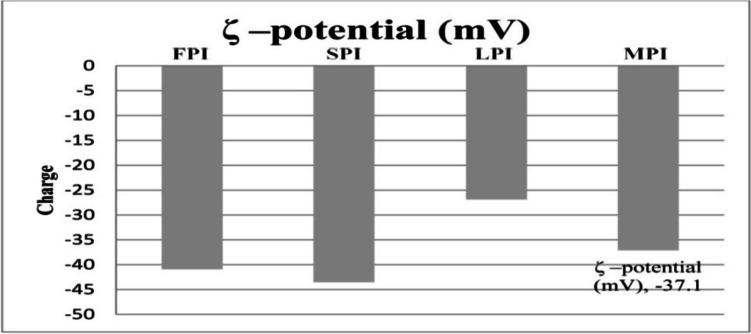
Zeta potential of FPI, SPI, LPI, and MPI; FPI: Faba bean protein isolate; SPI: Soybean protein isolate; LPI: Lupine protein isolate; MPI: Mixture protein isolate

Zeta potential is used to describe the electrostatic interactions between particles in solution, and its value is related to the suspended particle surface charge distribution. Protein particles typically stabilize a solution when their surface charges are high (above +30 mV or below −30 mV) whereas at surface charges between ±30 mV, protein–protein tend to be aggregated, resulting in low solubility and a propensity for precipitation [20]. The zeta potential values for the FPI, SPI, LPI, and MPI samples at pH 7.0 are shown in Fig. 2. A variance analysis revealed that all isolates were statistically distinct (*p* > 0.05). Compared to faba, mixed sample, or lupine (−40.9, −37.1, and − 26.9 mV, respectively), SPI exhibited a greater negative charge (−43.5 mV). Similarly, Johnston et al. [21] examined the surface charge of protein isolates from chickpea (CPI), faba bean (FPI), lentil (LPI), and soy (SPI) at pH 7.0. They discovered that all isolates had statistically identical values for CPI, FPI, LPI, and SPI, respectively, at −47.7, −46.4, −47.2, and − 44.3 mV, which are analogously in line with our findings. Meanwhile, Shi & Nickerson’s [22] FPI findings was comparable to ours (−41.8 mV), however, at pH 7, SPI in their investigation was shown to be less negative (−38.4 mV) than in our study [22]. Protein surface charge measurements are influenced by solvent conditions and surface electrochemical properties [23]. Therefore, in addition to extrinsic factors like sample preparation and protein extraction, intrinsic variables linked to cultivar changes, such as protein composition, conformation, and amino acid profile, may influence the variability of the results. Additionally, according to [24], protein isolates obtained via AE-IP showed a greater surface charge than those made using salt extraction. In agreement with Shi & Nickerson [22], the high surface charges of the examined legume protein isolates generally suggested excellent solubility in solution at pH 7.

### Differential Scanning Calorimetry (DSC)

**Fig. 3:**
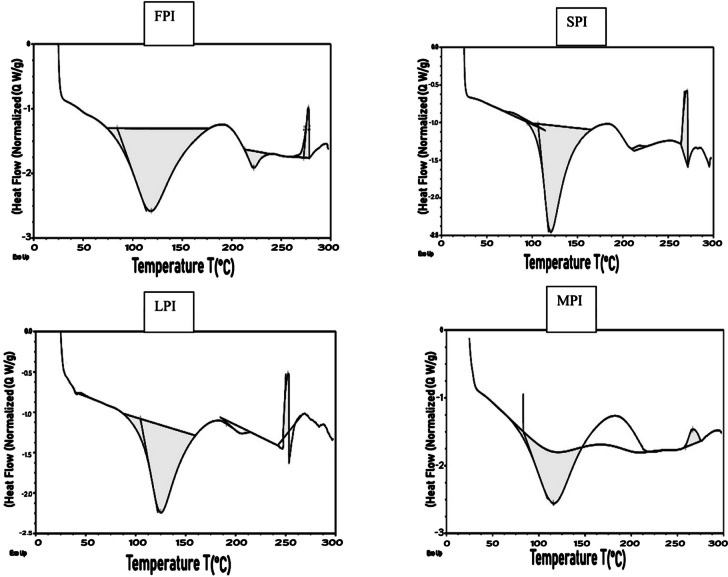
DSC thermograms of FPI (Faba bean protein isolate); SPI (soybean protein isolate); LPI (lupine protein isolate); MPI (mixture protein isolate)

The temperature transitions between 0 °C and 300 °C of the isolated plant proteins were investigated using DSC technique. As shown in Fig. 3, FPI displays two endothermic peaks which indicates the occurrence of multiple thermal transitions or events within the sample, the first peak was at 118.79 °C, and the second one was at 222.23 °C. However, SPI sample exhibited a peak at 120.69 °C that was noticeably longer.

Compared to the LPI sample, which showed a moderate endothermic peak with a maximum temperature around 124.86 °C, the MPI samples had more distinct patterns. The thermogram for the MPI sample showed an exothermic peak at 266.42 °C and a lowest endothermic peak at 115.54 °C. From 0 to 150 °C, nearly all four samples displayed identical enthalpies, and the thermograms revealed identical denaturation peaks. These results were consistent with those obtained by Ricci and his colleges [25] who examined pea, lentil, faba bean, chickpea and beans proteins and found that all samples finished analysis at about 150 °C [25]. However, at temperatures between 200 and 300 °C, MPI showed extreme peak stability which might be occurred due to increasing the moisture content compared to other samples [25]. Abugoch et al.’s [26] fluorescence experiments have already established the larger amount of denaturation [26]. The enthalpy differences reflect the extent of ordered structure of the globulins as the transition from native to denatured state took place. Some researchers returned this result to 11S globulin, as this fraction is the main storage protein. Thus, DSC technique has determined that proteins are denatured at pH 11, while extraction at pH 9 caused the highest degree of aggregation [27]. The observed values of temperature range depended on both protein origin and extraction pH [25].

### Microstructure

**Fig. 4:**
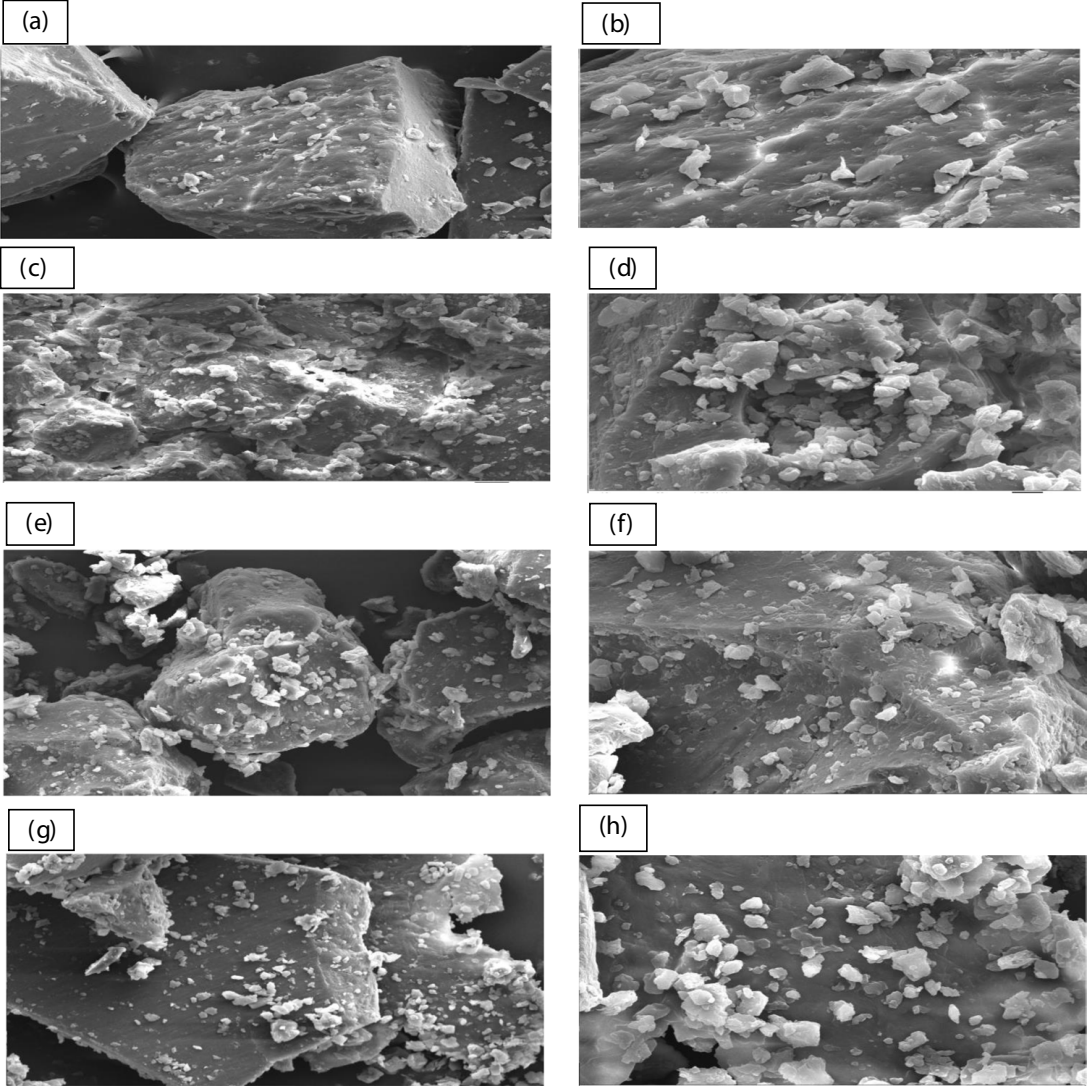
Microstructure of FPI [**a**, **b**], SPI [**c**, **d**], LPI [**e**, **f**] and MPI [**g**, **h**]; (**a**, **c**, **e**, and **g**), magnification 500; (**b**, **d**, **f**, and **h**), magnification1500

The microstructural characteristics of the FPI, SPI, LPI, and MPI are displayed in images obtained using a scanning electron microscope (SEM) at two different magnifications 500 and 1500 (Fig. 4). At a magnification of 500, it is clear to observe the angular edges on one side of the FPI sample and the spherical portion on the opposite side of the same particle, which amply demonstrates protein aggregation and the fact that some proteins naturally adopt a globular or spherical form as a component of their initial structure. In globular proteins, the polypeptide chain folds into a compact, spherical form that is maintained by several types of bonding interactions [28]. Meanwhile, Vogelsang-O’Dwyer [3] examined the microstructure of FPI and discovered that the particles had a smooth, shrunken appearance typical of spray-dried high protein powders. Differences in extraction techniques showed discrepancy in protein structure between our results and earlier studies [27]. SPI was magnified at 500 and 1500, revealing an uneven structure with bigger pores, random pore sizes, and small tiny fractions. Whereas, LPI showed a smooth surface covered by smaller fractions. These results agreed with those of Zhao et al. [29], who discovered that the former soybean protein microstructure lacked evenly spaced holes on the surface. On the other hand, the MPI sample had essentially similar angular edges to the FPI sample (magnification 500). Additionally, the surface of the particle was covered with extremely small portions as was previously observed in SPI and LPI.

### Rheological Properties

**Fig. 5:**
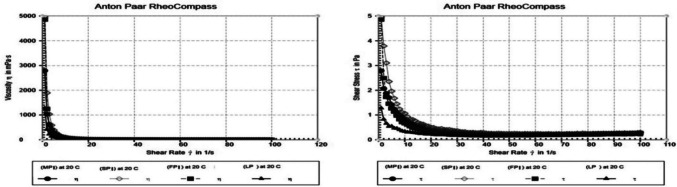
Flow and viscosity curves of the FPI, SPI, LPI and MPI protein isolates

Figure 5 displays some representative flow and viscosity curves for the protein isolates. The power law and viscosity ([mPas]) at a D = 50/S shear rate) from Table 2 were used to model flow curves. A non-Newtonian fluid with pseudoplastic flow was present and becomes more pronounced as *P* value decreases. Shear-thinning behavior is preferred in many food applications because it facilitates the flow, distribution, and application of protein dispersion. On the other hand, greater shear speeds leads to viscosity declining in all samples. Moreover, no differences were observed among all the protein dispersions, and all samples displayed the same slope of the viscosity. The viscosity of the dispersion lowers as the shear rate rises, improving process ability [30] These results were in agreement with those obtained by Rafe et al. [31] who found that the viscosity was decreased linearly with frequency demonstrating the shear thinning phenomenon.

**Table 2:** Parameters that characterize the viscosity and the flow curve

Sample	η [m. Pa.s]	P
FPI	3.7	0.8324
SPI	5.2	0.8755
LPI	3.1	0.8361
MPI	4.3	0.8444

η [mPas] = Apparent viscosity at D = 50/S, p = Flow behavior index, *FPI* Faba bean protein isolate, *SPI* Soybean protein isolate, *LPI* Lupine protein isolate, *MPI* Mixture protein isolate

**Fig. 6:**
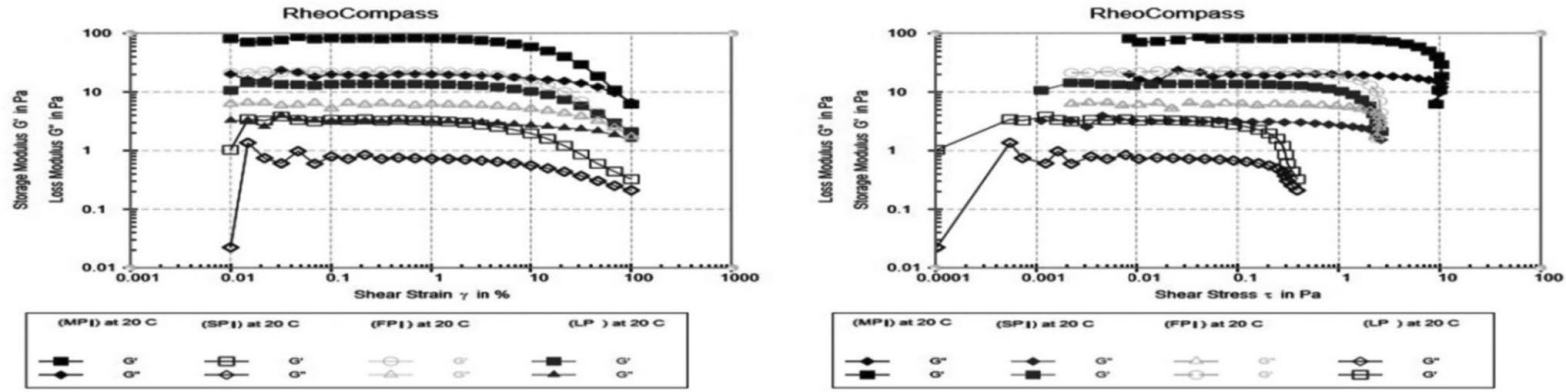
Amplitude sweeps (G’ and G”) for FPI, SPI, LPI, and MPI protein isolates

To determine the impact of applying various shear stress and strain pressures on the storage modulus (G’) and loss modulus (G”) of plant-based protein dispersions (FPI, SPI, LPI, MPI), an oscillatory test was conducted. The same linear curves and deterioration trend were observed in all samples (Fig. 6 and Table 3). G’ and G” values were measured with a clearly defined maximum point up to a critical point, which was followed by a sharp fall as shear stress and strain increased. The fact that G’ (storage modulus) was larger than G” (loss modulus) over the full shear stress range in all samples under investigation suggests that protein dispersion samples have a high capacity to store energy [32]. The MPI dispersion exhibited the highest storage modulus (G’) values with 88.419 Pa at 0.042565 Pa shear stress and decreased gradually to 82.321 Pa at 0.85294 Pa shear stress, then declined to 6.1694 Pa at a shear rate of 9.1905 Pa. A higher storage modulus denotes a more solid-like behavior, with increased structural integrity and resistance to deformation. These results were almost the same with those obtained by previous researchers who suggested that protein isolate suspensions with just acid denature exhibited high elasticity [33].

**Table 3:** Parameters for the linear viscoelastic range

Sample		FPI	SPI	LPI	MPI
G’ point (storage modulus) Pa	Maximum	22.757	14.337	3.8149	88.419
	Critical	13.04	12.178	2.9864	82.321
	Minimum	1.6161	2.1058	0.32472	6.1694
G” point (loss modulus) Pa	Maximum	6.4974	3.9182	1.3618	23.612
	Critical	4.7367	2.9071	0.70473	20.087
	Minimum	1.7123	1.5279	0.20952	6.7234

*FPI* Faba bean protein isolate, *SPI* Soybean protein isolate, *LPI* Lupine protein isolate, *MPI* Mixture protein isolate

## Conclusion

In this research, the innovation of a mixed combination from different plant-based protein sources may offer proteins with superior qualities to those of a single plant protein and resemble nutritional and industrial features as animal proteins. Legumes are increasingly being explored as alternatives to animal protein, as it is not only being adaptable to harsh conditions and low-input farming but also high nutritional value. Thus, protein isolates (faba beans, lupine, soybeans, and their combination) from a dietary and industrial standpoint were characterized. The combined sample revealed one of the highest protein levels among the legumes examined and a superb amino acid profile, with a better amino acid score comparable to that of animal-based protein, which is introduced as a vegan alternative. Additionally, the results indicated that its physiochemical, structural, and thermal qualities were exceptional due to high essential amino acids content, high surface charges resulted in excellent solubility and a stable curve when DSC test was conducted.

### Supplementary Information

ESM 1 (DOC 76 kb)

## Data Availability

All data generated or analyzed for this study are included in this published article (and its Supplementary Information file).

## Abbreviations

FPI: Faba bean protein isolation
LPI: Lupine protein isolation
MPI: Mixture protein isolation
SPI: Soybean protein isolation
HPLC: High-performance liquid chromatography
SEM: Scanning electron microscopy
DSC: Differential scanning calorimetry
ZP: Zeta-potential
G’: Storage modulus
G”: Loss modulus

## References

[1] Green H, Broun P, Cook D (2018). Healthy and sustainable diets for future generations. J Sci Food Agric.

[2] Lizarazo CI, Lampi A, Liu J (2015). Nutritive quality and protein production from grain legumes in a boreal climate. J Sci Food Agric.

[3] Vogelsang-O’Dwyer M, Bez J, Petersen IL (2020). Techno-functional, nutritional and environmental performance of protein isolates from blue lupin and white lupin. Foods.

[4] Mínguez MI, Rubiales D (2021) Faba bean. In: Crop physiology case histories for major crops. Elsevier, pp 452–481. 10.1016/B978-0-12-819194-1.00015-3

[5] Batista AP, Portugal CAM, Sousa I (2005). Accessing gelling ability of vegetable proteins using rheological and fluorescence techniques. Int J Biol Macromol.

[6] Cai R, McCurdy A, Baik B (2002). Textural property of 6 legume curds in relation to their protein constituents. J Food Sci.

[7] Mohanan A, Nickerson MT, Ghosh S (2020). Utilization of pulse protein-xanthan gum complexes for foam stabilization: the effect of protein concentrate and isolate at various pH. Food Chem.

[8] Day L (2013). Proteins from land plants–potential resources for human nutrition and food security. Trends Food Sci Technol.

[9] Wang H, Liu F, Ma H (2021). Associations between depression, nutrition, and outcomes among individuals with coronary artery disease. Nutrition.

[10] Boukid F, Castellari M (2022). How can processing technologies boost the application of faba bean (Vicia faba L.) proteins in food production?. eFood.

[11] Gorissen SHM, Crombag JJR, Senden JMG (2018). Protein content and amino acid composition of commercially available plant-based protein isolates. Amino Acids.

[12] Eckert E, Han J, Swallow K (2019). Effects of enzymatic hydrolysis and ultrafiltration on physicochemical and functional properties of faba bean protein. Cereal Chem.

[13] Felix M, Romero A, Carrera-Sanchez C, Guerrero A (2019). Assessment of interfacial viscoelastic properties of Faba bean (Vicia faba) protein-adsorbed O/W layers as a function of pH. Food Hydrocoll.

[14] Klupšaitė D, Juodeikienė G (2015). Legume: composition, protein extraction and functional properties. A review. Chem Technol.

[15] Kudełka W, Kowalska M, Popis M (2021). Quality of soybean products in terms of essential amino acids composition. Molecules.

[16] Mohsen SM, Fadel HHM, Bekhit MA (2009). Effect of substitution of soy protein isolate on aroma volatiles, chemical composition and sensory quality of wheat cookies. Int J Food Sci Technol.

[17] Nosworthy MG, Medina G, Franczyk AJ (2018). Effect of processing on the *in vitro* and *in vivo* protein quality of beans (Phaseolus vulgaris and Vicia Faba). Nutrients.

[18] Ayala-Rodríguez VA, López-Hernández AA, Lomelí ML-C et al (2022) Nutritional quality of protein flours of fava bean (*Vicia faba* L.) and *in vitro* digestibility and bioaccesibility. Food Chem X 14:100303. 10.1016/j.fochx.2022.100303 10.1016/j.fochx.2022.100303 PMC9018142 35450143

[19] Rushovich D, Weil R (2021). Sulfur fertility management to enhance methionine and cysteine in soybeans. J Sci Food Agric.

[20] Rodríguez-Ambriz SL, Martínez-Ayala AL, Millán F, Davila-Ortiz G (2005). Composition and functional properties of Lupinus campestris protein isolates. Plant Foods Hum Nutr.

[21] Johnston SP, Nickerson MT, Low NH (2015). The physicochemical properties of legume protein isolates and their ability to stabilize oil-in-water emulsions with and without genipin. J Food Sci Technol.

[22] Shi D, Nickerson MT (2022). Comparative evaluation of the functionality of faba bean protein isolates with major legume proteins in the market. Cereal Chem.

[23] Wongsagonsup R, Shobsngob S, Oonkhanond B, Varavinit S (2005). Zeta potential (ζ) analysis for the determination of protein content in rice flour. Starch-Stärke.

[24] Karaca AC, Low N, Nickerson M (2011). Emulsifying properties of chickpea, faba bean, lentil and pea proteins produced by isoelectric precipitation and salt extraction. Food Res Int.

[25] Ricci L, Umiltà E, Righetti MC (2018). On the thermal behavior of protein isolated from different legumes investigated by DSC and TGA. J Sci Food Agric.

[26] Abugoch LE, Romero N, Tapia CA (2008). Study of some physicochemical and functional properties of quinoa (Chenopodium quinoa Willd) protein isolates. J Agric Food Chem.

[27] López-Castejón ML, Bengoechea C, Díaz-Franco J, Carrera C (2020). Interfacial and emulsifying properties of quinoa protein concentrates. Food Biophys.

[28] Zhang R, Han Y, Xie W (2022). Advances in protein-based nanocarriers of bioactive compounds: from microscopic molecular principles to macroscopical structural and functional attributes. J Agric Food Chem.

[29] Zhao X, Zhu H, Zhang B (2015). XRD, SEM, and XPS analysis of soybean protein powders obtained through extraction involving reverse micelles. J Am Oil Chem Soc.

[30] Ralston BE, Osswald TA (2008). Viscosity of soy protein plastics determined by screw-driven capillary rheometry. J Polym Environ.

[31] Rafe A, Seddighi R, Mousavi M, Bastan E (2023). Dynamic rheological properties of sesame protein dispersions. Legum Sci.

[32] Giri SK, Tripathi MK, Kotwaliwale N (2018). Effect of composition and storage time on some physico-chemical and rheological properties of probiotic soy-cheese spread. J Food Sci Technol.

[33] Bi C, Chi S, Hua Z (2020). Rheological properties and fractal-rheology analysis of peanut protein isolate suspension. Int J Agric Biol Eng.

