# Supplementary material for: Looking for a Novel Vegan Protein Supplement from Faba Bean, Lupine, and Soybean: a Dietary and Industrial Standpoint
Source: Plant Foods Hum Nutr. 2023 Dec 7;79(1):90–7. doi: 10.1007/s11130-023-01125-y (PMC10891211; doi:10.1007/s11130-023-01125-y)
Supplement: Supplementary file 1 — (DOC 76 kb) [file 11130_2023_1125_MOESM1_ESM.doc]

# **Supplementary Material**

# “Looking for a Novel Vegan Protein Supplement from Faba Bean, Lupine, and Soybean: A Dietary and Industrial Standpoint”

# **Hend A. Hamed^1^, Walaa Kobacy^2^, Elsayed A. Mahmoud^2^, Mennatallah M. A. El-Geddawy^3^**

# **^1^**Sohag University, Faculty of Agriculture, Horticulture Department, Sohag, 82524, Egypt.

# **^2^** Sohag University, Faculty of Agriculture, Food Science & Nutrition Department, Sohag, 82524, Egypt.

# **^3^**Assuit University, Faculty of Agriculture, Food Science & Technology, Department, Assuit, 71526, Egypt.

# **Abbreviations**

# **FPI** Faba beans Protein Isolation

# **SPI** Soy beans Protein Isolation

# **LPI** Lupine Protein Isolation

# **MPI** Mixture Protein Isolation

# **Materials and Methods:**

# **Field experiment and plant material preparation**

Cultivars of faba bean (Misr1), lupine (Baladi), and soybean (Giza 111) were cultivated at the experimental farm, Assuit University, Egypt, in 2021. Table 1 shows the soil type, mechanical, and chemical properties, and minimum and maximum temperature of the cultivation site. Cultivation of faba bean (Misr1) and lupine was on 1^st^ November and on 1^st^ May for soybean. Plot sizes were 2.5 m × 6 m. Row spacing was 25 with 5 cm between plants. Plots were fertilized with recommended doses of N, P, and K. At maturity, seed samples were harvested.

**Table. 1.** Summary description of the experimental site.

| **Parameters** | | **Value** |
| --- | --- | --- |
| **Location** | **Latitude** | 27.2061°N |
|  | **Longitude** | 31.1622°E |
| **Mechanical analysis** | **Sand** | 10–20% |
|  | **Slit** | 30–40% |
|  | **Clay** | 50–60% |
| **Chemical analysis** | **Organic matter** | 6–12% |
|  | **pH** | 3.8–4.8 |
| **1^st^ May for soybeans** | **Temperature** | 37.8ºCmaximum  21.6ºCminimum |
|  | **Humidity** | 24% |
| **1^st^ November for faba beans and lupine** | **Temperature** | 30ºC maximum  18ºC minimum |
|  | **Humidity** | 41% |

# **Protein Isolation**

Alkaline solution and isoelectric precipitation (AE‐IP**)** [1] were used to extract the protein isolates from faba bean, soybean, and lupine samples. However, some additional steps have been added to the samples to lessen antinutrient elements and enhance the extraction efficiency and nutritional value of the protein samples. Therefore, seeds were steeped in distilled water for 12 h and then allowed to germinate for 48 h. Then they were dried for 36 h at 60 ºC in the oven. Dry germinated seeds of faba bean, soybean and lupine were ground. This step is followed by sieving through a 50-mesh sieve. The flour obtained from the three samples was defatted separately in a Soxhelt apparatus to reduce the high-fat content. The resultant flours were dispersed in water in a 1:5 (w/v) ratio, and the pH of the suspension was adjusted to 9.0 with 0.1N NaOH. The mixture was stirred at room temperature for 20 min. The insoluble matrices were separated by centrifugation (4000 g/20 min, room temperature) and discarded. The supernatant was adjusted to 4.0 pH with 1 N HCl and stirred at room temperature for 20 min. The mixture was centrifuged (4000 g/20 min., room temperature), and the protein pellet dried. Protein isolates from faba bean, soy bean and lupine (FPI, SPI, and LPI) were labeled and then the mixed sample (MPI) was prepared by combining (1:1:1) of the three previous protein isolates. Then they were kept at -80ºC for further analysis.

# **Crude protein**

The nitrogen (N) content of the samples was determined according to [2] using the Kjeldahl method. N*6.25 was used to compute total protein.

# **Amino Acid Analysis**

Amino acids were determined by precolumn derivatization with diethyl ethoxymethylenemalonate and reversed-phase high-performance liquid chromatography (HPLC) with spectrophotometric detection at 280 nm according to Alaiz et al. [3]. The HPLC system consisted of a Merck-Hitachi L-6200A pump (Merck, Darmstadt, Germany) with a Rheodyne 7725i injector with a 20 μL sample loop, a Merck-Hitachi L-4250 UV-vis detector, and a Merck-Hitachi D-2500 chromato-integrator. Separation of derivatives was attained using a Nova-Pack C18 (300 × 3.9 mm id, 4 μm particle size; Waters, Milford, MA). Sample preparation were held according to [4] as well as chromatographic conditions.

# **Differential Scanning Calorimetry (DSC)**

DSC analyses were carried out at 20 ⁰C min^−1^. A PerkinElmer DSC 8500 differential scanning calorimeter with an Intercooler III chilling system was used to conduct the calorimetric experiments. According to the protocol for standard DSC, the instrument was calibrated in temperature and energy using high-purity standards (indium, naphthalene, and cyclohexane). Aluminum DSC pans that were sealed, held samples weighing approximately 10 mg. Dry nitrogen was used as the purge gas at a rate of 30 mL min^−1^ [5].

# **Zeta-potential measurements**

Zeta Potential of the samples was determined using Nano Particle Zeta Potential Analyzer (Nano PIUS-3, Mike Instruments, New York, NY, USA). It is measured under the condition of pH = 7 and protein concentration of 2% (w/v) [6].

**Microstructural analysis**

The Surface morphologies of the lyophilized FPI, SPI, LPI, and MPI protein isolate samples were characterized using scanning electron microscopy (SEM) and the mapping method (ZEISS). SEM was performed on dried protein samples, which were stuck on aluminum stubs with double sticky tape coated with10nm gold. At magnifications of 500 x and 1500 x, micrographs of the sample microstructure were captured [7].

**Rheology measurements**

For rheological studies, protein isolates were dispersed in citrate phosphate buffer (15 w/w% protein, pH 8) using a stirrer for 1 h at room temperature. Rheological studies were performed in a UDS 200 rheometer (PAAR PHYSICA) at 20 8C using a cone-plate system (cone diameter 75 mm, angle 18). The edge of the cone was covered by a low-viscosity silicon oil to avoid evaporation of water from the sample. After introducing the protein sample into the rheometer, the sample was sheared at 1 rpm for 5 min to ensure an equal ªsample historyº. A time of standing of 10 min was kept between each measuring interval. After that, the flow curve was recorded (shear rate D = 50 1/s) and characterized by the flow index, p, calculated according to the power law and the apparent viscosity at D = 50 s±1 [8].

The oscillatory test was studied by increasing the shear rate and shear stress at a rate of 1 degree/min and measuring the loss (G'') and storage modulus (G') (f = 1 Hz, c = 5%). The mechanical properties (linear viscoelastic range, maximum shear stress) of the protein dispersions were tested by an amplitude sweep (c = 1%±100%, f = 1 Hz) [9].

**Statistical analysis**

Experiments were carried out in triplicate, and the values are presented as the mean ± Standard deviation. A significant difference of p < 0.05 was taken in an SPSS 22.0 one-way analysis of variance (ANOVA) [10].

# **References**
